# Five-year dementia prediction and decision support system based on real-world data

**DOI:** 10.3389/fnagi.2025.1670609

**Published:** 2025-09-30

**Authors:** Themis P. Exarchos, George A. Dimakopoulos, Konstantinos Lazaros, Marios Krokidis, Aristidis Vrahatis, Gerasimos Grammenos, Antigoni Avramouli, Konstantina Skolariki, Roy Adams, Vasiliki Mahairaki, Esther S. Oh, Jeannie Leoutsakos, Paul B. Rosenberg, Constantine G. Lyketsos, Panagiotis Vlamos

**Affiliations:** 1 Bioinformatics and Human Electrophysiology Laboratory, Department of Informatics, Ionian University, Corfu, Greece; 2 Institute of Digital Biomedicine, Ionian University Research and Innovation Center, Corfu, Greece; 3 Johns Hopkins University School of Medicine, Baltimore, MD, United States; 4 Johns Hopkins Bayview Medical Center, Baltimore, MD, United States

**Keywords:** dementia prediction, Alzheimer's disease, electronic health records, clinical study, cognition, patient-level prediction, real-world data, risk prediction

## Abstract

**Introduction:**

This work presents a machine learning (ML) based risk prediction model for Alzheimer's disease and related dementias, utilizing real-world electronic health record (EHR) clinical data. While significant research has been conducted on dementia risk prediction, most studies rely on volunteer-based research cohorts rather than real-world clinical data. Using raw EHR data offers more realistic insights but poses challenges due to the extensive effort required to convert real-world EHR clinical data into a decision support system for daily clinical use.

**Methods:**

The dataset consists of a high-volume, ten-year export of raw EHR data from Epic, the Johns Hopkins (JH) Health System. In this study, we utilized multimodal JH EHR data to develop a patient-based model to predict dementia onset over a five-year period. The interpretable binary classification model identified prognostic rulesets for dementia based on clinical characteristics.

**Results:**

The model achieved a mean test accuracy of 0.722 (95% CI: 0.722–0.723) and an AUROC of 0.795 (95% CI: 0.794–0.795) using 5-fold cross-validation across different sample subsets.

**Discussion:**

Recognizing that neurodegenerative diseases are often driven by multiple contributing factors rather than a single cause, we identify risk pathways by leveraging multimodal data and modeling their combined effects, leading to accurate dementia predictions and improved clinical interoperability.

## 1 Introduction

Electronic Health Records (EHRs) have been implemented in over 90% of hospitals and clinics across the United States, creating a vast repository of patient data that serves as a valuable source of real-world data (RWD) for research (Office of the National Coordinator for Health Information Technology, 2022; Kim et al., 2023). Unlike traditional registries or insurance claims databases, which often experience significant time lags, EHR systems continuously capture up-to-date, longitudinal data generated during routine clinical care. These records encompass both structured data—such as coded diagnoses, laboratory results, prescribed medications, and demographic information—and unstructured data, including physician notes, discharge summaries, and patient history narratives. The immediacy and depth of EHR-derived RWD provide significant opportunities for developing predictive models and generating real-world evidence in healthcare. One major benefit of training models on real-world clinical data is enhanced generalizability (Bakouny and Patt, 2021; Rashidisabet et al., 2023; Amrollahi et al., 2022). By learning from diverse and heterogeneous patient populations, these models better reflect the variability encountered in clinical practice, increasing robustness and reliability when applied to new settings. However, RWD require thorough bias analysis, as biases can emerge at various stages, including data generation, extraction, and modeling. Realizing the full potential of RWD requires overcoming several critical challenges (Bastarache et al., 2022; Collins and Tabak, 2014). EHR data are inherently heterogeneous, often unstructured, and frequently incomplete, necessitating advanced techniques for preprocessing, standardization (Kim and Min, 2025), integration, and effective learning. Challenges such as missing values (Ren et al., 2024), irregular data sampling over time (Chauhan et al., 2024), and systematic biases in data collection (Al-Sahab et al., 2024) can significantly impact model performance if not properly managed. Recent research have shown promising results on identifying Alzheimer's disease and related dementias at earlier stages using machine learning methods on EHR data. Many studies have shown that both structured and unstructured clinical data, such as medication histories, clinical narratives, and behavioral patterns can be mined to detect indicators of cognitive decline (Ford et al., 2019; Jammeh et al., 2018). Some approaches have proposed passive digital signatures extracted from longitudinal EHR data to determine dementia risk years before onset symptoms (Boustani et al., 2020), while others have improved predicting accuracy using polygenic risk scores, behavioral symptoms and socioeconomic factors withing large scale population datasets (Gao et al., 2023; Li et al., 2023). Another study applied label learning on large-scale claims and EHR data, achieving strong predictive performance for incident AD within 2 years by addressing diagnostic uncertainty inherent in administrative datasets (Nori et al., 2019). Further work, has shown the importance of multimodal models that combine EHR data with environmental and social factors on capturing disease heterogeneity (Tang et al., 2024). These studies also highlight the importance of temporal dynamics and functional decline in improving prediction over 1 to 5 year windows as well as the growing use of explainable machine learning methods to identify key predictors such as sleep apnea, disorientation, depressive symptoms and comorbid conditions (Akter et al., 2025).

## 2 Materials and methods

### 2.1 Source of data

**Figure 1:**
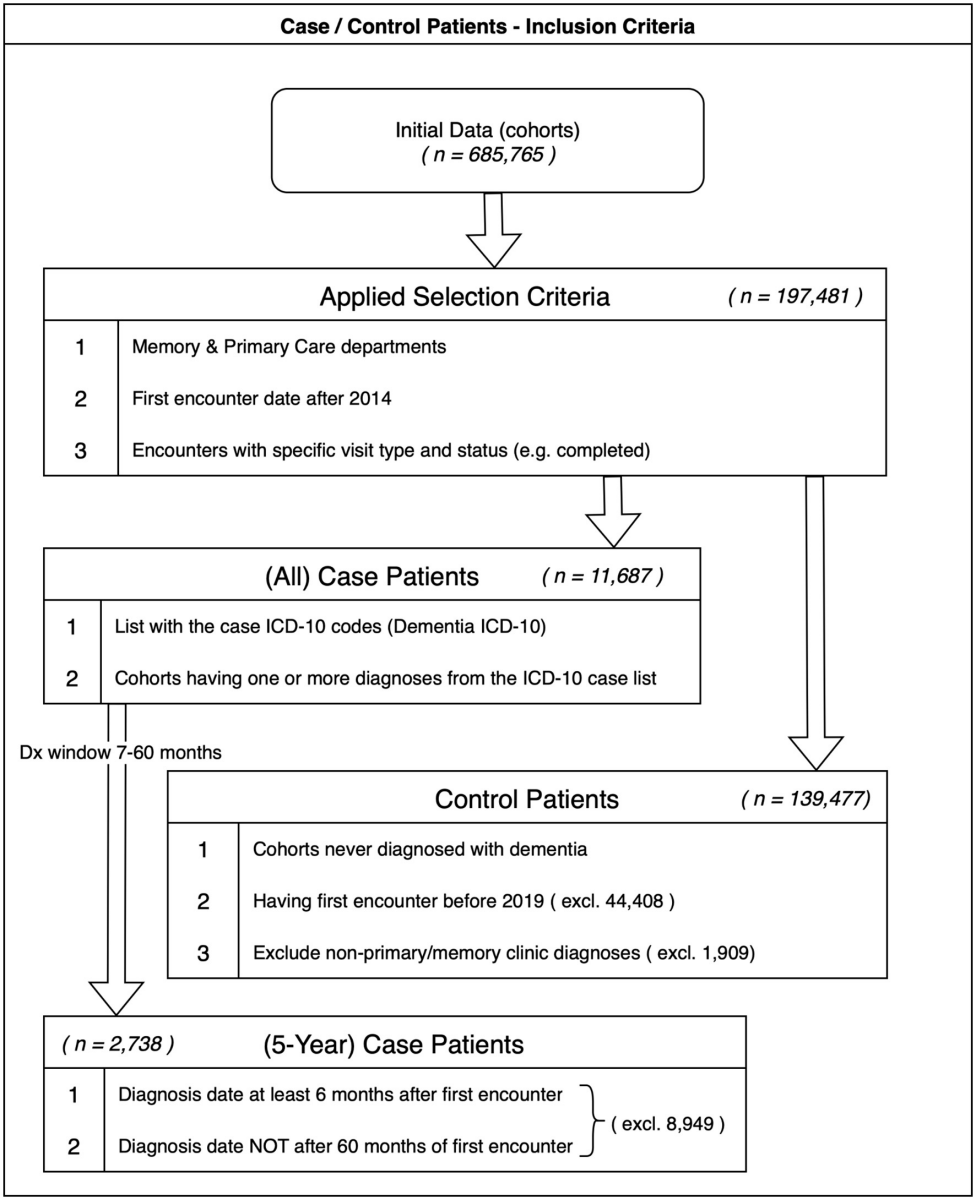
Inclusion criteria.

We analyzed RWD from the Johns Hopkins Health System EHR (Epic). The EHR initially contained 685,765 cohorts. Applying specific inclusion criteria, such as patient profile constraints and visit attributes related to primary care, memory care, and completion status, refined to a baseline of 197,481 patients. This final group includes individuals with 10 years of EHR data, spanning from January 1, 2014, to December 31, 2023, from both primary care and memory clinics within the Johns Hopkins (JH) health system (Figure 1). The memory clinic includes outpatients of the Johns Hopkins Memory and Alzheimer's Treatment Center (JHMATC) in Baltimore Maryland, USA with at least one visit between 2014 and the end of 2023. The primary care data includes all outpatients with at least one encounter during the same period within Johns Hopkins primary care clinics. The Johns Hopkins primary care clinics, located across the Maryland/DC area, provide a range of primary care services. Table 1 summarizes the demographic and clinical characteristics of the study population. Table 1 outlines the demographic and clinical characteristics of the study cohort. After the initial selection process for CI stage classification, the final sample consisted of 142,175 patients, with 139,437 (98.1%) classified in the control group and 2,738 (1.9%) classified in dementia. The mean age increased across the groups, from 58.5 years in the control group to 74.7 years in the dementia group. It should be noted that our dataset combines primary care data, which covers a broader age range, and memory clinic data, where patients tend to be older and the age range is narrower. This reduces strict comparability because age is a major risk factor for dementia and some of the observed differences may therefore reflect age-related effects rather than disease-specific characteristics. However, since age is not included as a model feature, this limitation should be carefully considered when interpreting the results. Future studies should aim to include age-matched controls or apply statistical methods to minimize the influence of age on the findings.

**Table 1:** Sample characteristics.

**Characteristics**	**Total *N* = 142,175^a^**	**Cognitive impairment stage**	
		**Control** ***N*** = **139,437 (98.1%)**	**Dementia** ***N*** = **2,738 (1.9%)**
Age^b^ (Mean and SD)	58.8 (11.1)	58.5 (10.9)	74.7 (10.0)
< 65	101,283 (71.2%)	100,908 (72.4%)	375 (13.7%)
65−74	26,838 (18.9%)	25,963 (18.6%)	875 (32.0%)
75−84	10,891 (7.7%)	9,820 (7.0%)	1,071 (39.1%)
85+	3,163 (2.2%)	2,746 (2.0%)	417 (15.2%)
**Sex**			
Female	81,861 (57.6%)	80,125 (57.5%)	1,736 (63.4%)
Male	60,314 (42.4%)	59,312 (42.5%)	1,002 (36.6%)
**Race**			
White	91,072 (64.1%)	89,284 (64.0%)	1,788 (65.3%)
Black	35,223 (24.8%)	34,511 (24.8%)	712 (26.0%)
Asian	2,101 (1.5%)	2,053 (1.5%)	48 (1.8%)
Other	5,331 (3.7%)	5,242 (3.8%)	89 (3.3%)
Unknown	8,448 (5.9%)	8,347 (6.0%)	101 (3.7%)
**Ethnicity**			
Hispanic	2,280 (1.6%)	2,236 (1.6%)	44 (1.6%)
Other	139,821 (98.3%)	137,127 (98.3%)	2,694 (98.4%)
Unknown	74 (0.1%)	74 (0.1%)	−

^a^Demographic data is unavailable for 40 of the 142,215 cohorts.

^b^Age is calculated from the date of birth at the patient's first visit to JH EHR.

Our analysis was based on ICD-10 coding to identify clinical diagnoses and comorbidities. Since ICD-10 was implemented on October 1, 2015, a small number of diagnoses were not captured due to the lack of consistent ICD-10 coding prior to its adoption. A case-control binary classification prediction target was estimated at the patient level, determining whether patients (cohort IDs) were ever diagnosed with any form of dementia during the study period, based on the dementia-related ICD-10 codes outlined in the Table 2. In this study, ICD-10 codes from the sample were mapped to disease categories using the International Classification of Diseases, 10th Revision (ICD-10), a globally recognized standard for coding diseases and health conditions maintained by the World Health Organization (WHO) (World Health Organization, 1992).

**Table 2:** Primary ICD-10 codes for dementia and related conditions.

**Category**	**Description**	**Patients (*N* = 3,688)^c^**
G30.*x*^a^	Alzheimer's disease (includes early/late onset, atypical, unspecified)	892
G31.84	Mild cognitive impairment, so stated (pre-dementia stage)	1,025
G31.83	Neurocognitive disorder with lewy bodies	56
G31.0	Frontotemporal dementia	−
F01.*x*	Vascular dementia	334
F02.*x*^b^	Dementia in other diseases (e.g., Parkinson's, Pick's, Huntington's)	144
F03.*x*	Unspecified dementia (used when cause is unknown or not documented)	1,237

^a^The (*x*) indicates all subcodes are included (e.g., F01.5, F01.A, F01.B, etc.).

^b^F02.*x* codes are used with other primary disease codes like G20 (Parkinson's), G23.1 (PSP), etc.

^c^Several patients were diagnosed with multiple dementia-related ICD-10 codes.

### 2.2 Cases and controls

In this study, time-stamped EHR records are transformed into a structured, tabular format, where each patient is represented by a set of features (predictors) and class labels assigned based on inclusion criteria, indicating whether the outcome occurs within the specified risk period (Shickel et al., 2018; Ferrao et al., 2017). The initial cohort collection process in Figure 1 included of 197,481 patients gathered from JHMATC and JHCP clinics, at their first encounter recorded in the EHR after January 1, 2014 and before December 31, 2023. To be included, encounters were required to have a valid and completed encounter type such as office visit, clinical support, video visit or follow-up visit. The process of classifying the patients into case and control groups is outlined below, resulting initially in 11,687 cases and 139,477 controls. As additional assessments are typically required before a final diagnosis of dementia can be made during a subsequent encounter, cases were included if their diagnosis was documented at least 6 months and at most 5 years after their first visit. Following this inclusion process for case cohorts, 2,738 case-labeled patients remain in the dataset, out of the 11,687 case patients selected in the initial phase (Table 3). Similarly, we assigned the control label to patients from the initial selection (197,481) if the patient never received a dementia diagnosis (i.e., the subject is not included in the case list). Of the resulting controls, 1,909 were excluded because they received dementia diagnoses outside of JHMATC or JHCP clinics. To increase the likelihood that control patients remained in the control group for at least five years, we imposed an additional constraint requiring their first encounter to have occurred before January 1, 2019. Although dementia often develops over extended periods, previous studies have demonstrated that a 5-year window is sufficient to capture a substantial proportion of incident cases and is commonly used in prognosis and prediction modeling. Extending the timeframe to 7–10 years would substantially limit the eligible cases in our cohort, as the observation period spans 2014 to the end of 2023.

**Table 3:** Creating collection of labeled data.

**Selection**	**Cohorts**
Initial selection	685,765
Apply selection criteria	197,481
Control—first visit < 2019	**139,477**
Control—first visit ≥2019	44,408
Exclude (controls) with non-primary/memory clinic dementia diagnoses	1,909
Dementia (All)	11,687
Dementia—first diagnosis between 7 and 60 months	**2,738**
Dementia—first diagnosis within the first 6 months or after 60 months	8,949

Baseline cohorts : 139,477 + 44,408 + 1,909 + 11,687 = 197,481.

Cohorts included in the final dataset : 139,477 + 2,738 = **142,215**. Numbers in bold indicate the control and disease cohorts retained in the final dataset.

Mitchell and Shiri-Feshki (2009) conducted a meta-analysis of 41 inception cohort studies of individuals with mild cognitive impairment (MCI). They reported an annual conversion rate (ACR) of 6.7% for progression from MCI to dementia, 6.5% specifically for Alzheimer's disease, and 1.6% for vascular dementia. These rates imply that over a 5-year follow-up window, a substantial proportion of individuals with MCI will progress to dementia—typically in the range of 25–35% or higher, depending on the cohort and diagnostic criteria. This evidence supports the adequacy of a 5-year observation window, as it is long enough to capture a meaningful number of incident dementia cases, making it a standard timeframe in prognostic and predictive modeling studies. Thus, while we acknowledge that dementia progression can extend beyond 5 years, our design ensures diagnostic stability of the control group, preserves sufficient sample size.

However, we acknowledge that the additional constraint requiring control patients' first encounter to occur before January 1, 2019 does not ensure complete follow-up, particularly for memory clinic patients, many of whom live out of state and may not be consistently captured in the EHR throughout the entire period.

### 2.3 Parameters

**Figure 2:**
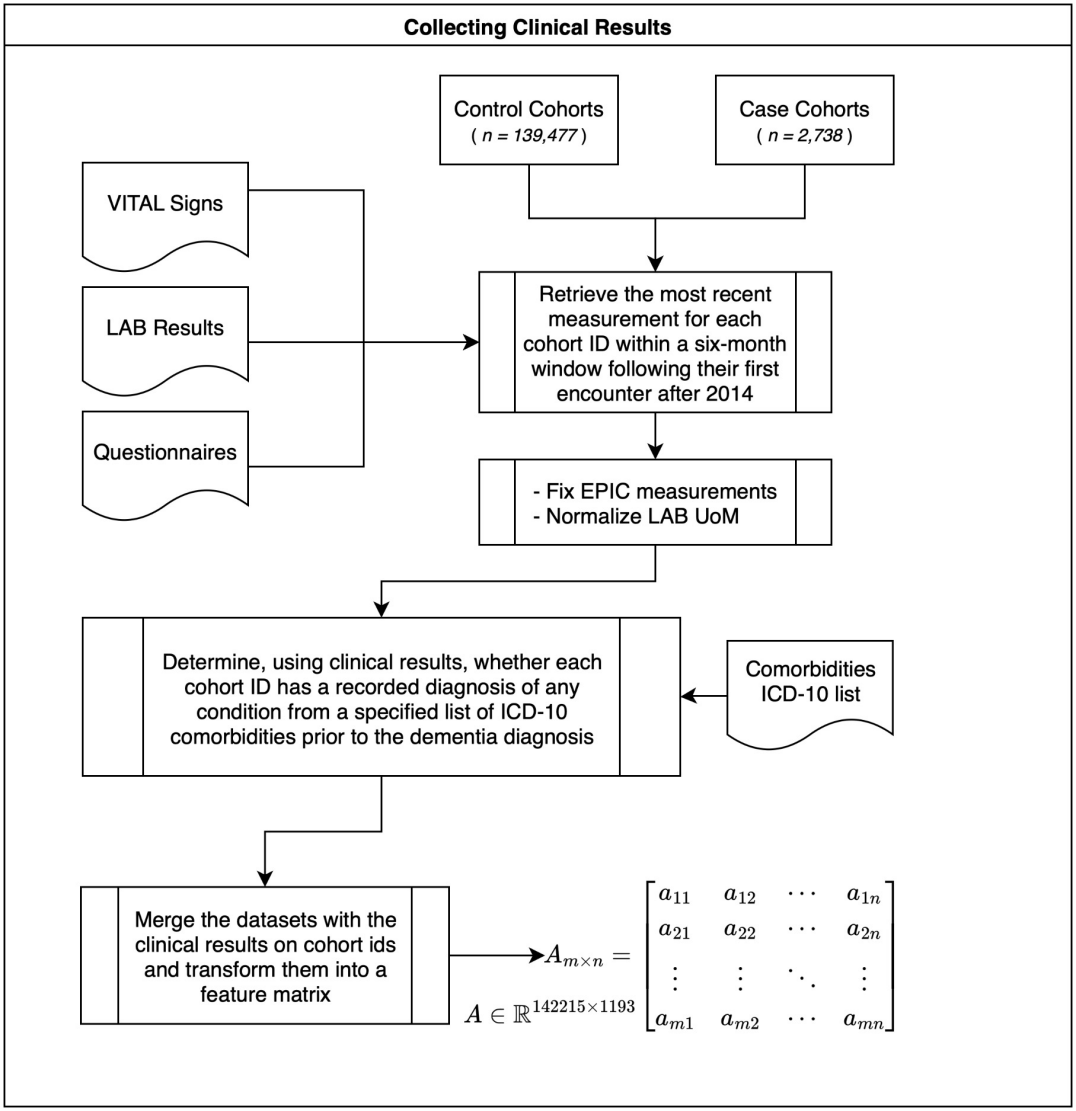
Collecting clinical measurements.

After assigning class labels to patients based on their encounter and diagnosis profile, the next step was to analyze the EHR data to extract the set of their medical covariates. The final multimodal dataset includes features from clinical measurements, vital signs, laboratory test results, and cognitive test questionnaire scores. The extracted measurements for both case and control patients included data from their (earliest) first recorded visit and a 6-month window following that particular initial encounter. With the 6-month window, we ensure that a broad range of measurements is collected, thereby minimizing missing values for each feature. For repeated measurements of the same feature, the most recent measurement is considered as the most accurate (Figure 2). While real-world data are invaluable for their realistic outcomes, working with such data presents significant challenges, particularly in transforming raw clinical data into a structured, machine learning-ready format (Kim and Min, 2025). Measurement fields in observational databases are often stored as strings, increasing the likelihood of typographical errors. Additionally, some values, such as blood pressure readings, are recorded as string formats of type systolic/diastolic. To standardize these measurements, blood pressure values were converted into a single numerical metric: the Mean Arterial Pressure (MAP) (DeMers and Wachs, 2025). For instance, a recorded value of “130/86” is replaced with the value of 100.66 which is its MAP equivalent. This conversion is labeled as “Fix EPIC measurements” in Figure 2. Likewise, laboratory measurements in the source data may appear in different units of measurement, necessitating a thorough unit conversion process to align each marker with a standard reference unit of measurement. Instead, we normalized measurements using the Normal Reference Range (NR) for each marker, adjusting lab results relative to the specific Upper and Lower Limits of Normal (ULN and LLN) as defined for each marker by the automated laboratory systems. This method offers several advantages over traditional unit conversions by eliminating dependency on measurement units and reducing the risk of UoM conversion errors. Instead, each lab measurement is documented alongside its normal reference range, which is present in 94% of records in the Johns Hopkins (JH) EHR system. Moreover, another key advantage of this method is that it makes minimal assumptions, relying solely on the source data (the actual measurement and its normal reference range) regardless of the unit of measurement. This enhances the ability to perform standardized measurement comparisons across diverse laboratory systems as well as specimen sources, such as blood and urine. In addition to vital signs, laboratory test results, and cognitive test questionnaires, we incorporated diagnoses of comorbidities (Figure 2). To identify the presence of comorbidity diagnoses, the EHR database was searched for ICD-10 codes associated with specific comorbidity categories. For case patients, comorbidity diagnoses were recorded before the diagnosis of dementia. We assumed that, if a diagnosis is not recorded in the EHR system, it does not exist. The list of comorbidity ICD-10 codes is derived from the International Classification of Diseases catalog (World Health Organization, 1992). Furthermore, we excluded blocks related to accidents or abnormal findings on examinations that did not lead to a diagnosis. Moreover, to prevent target leakage, we excluded from the comorbidities catalog the blocks (features) containing diagnoses that overlap with parts of the target ICD-10 codes [e.g., DISEASES OF THE NERVOUS SYSTEM (G30–G32)] or involve a diagnosis related to cognition [e.g., SYMPTOMS AND SIGNS INVOLVING COGNITION PERCEPTION EMOTIONAL STATE AND BEHAVIOR (R40–R46)]. The final step in collecting clinical measurements (Figure 2) involved merging all features, including clinical measurements (vital signs, laboratory results, and cognitive assessment scores) with the comorbidities diagnoses, into a unified table. This table was transposed into a matrix consisting of 1,193 features as columns and 142,215 rows (observations).

### 2.4 Preprocessing

**Figure 3:**
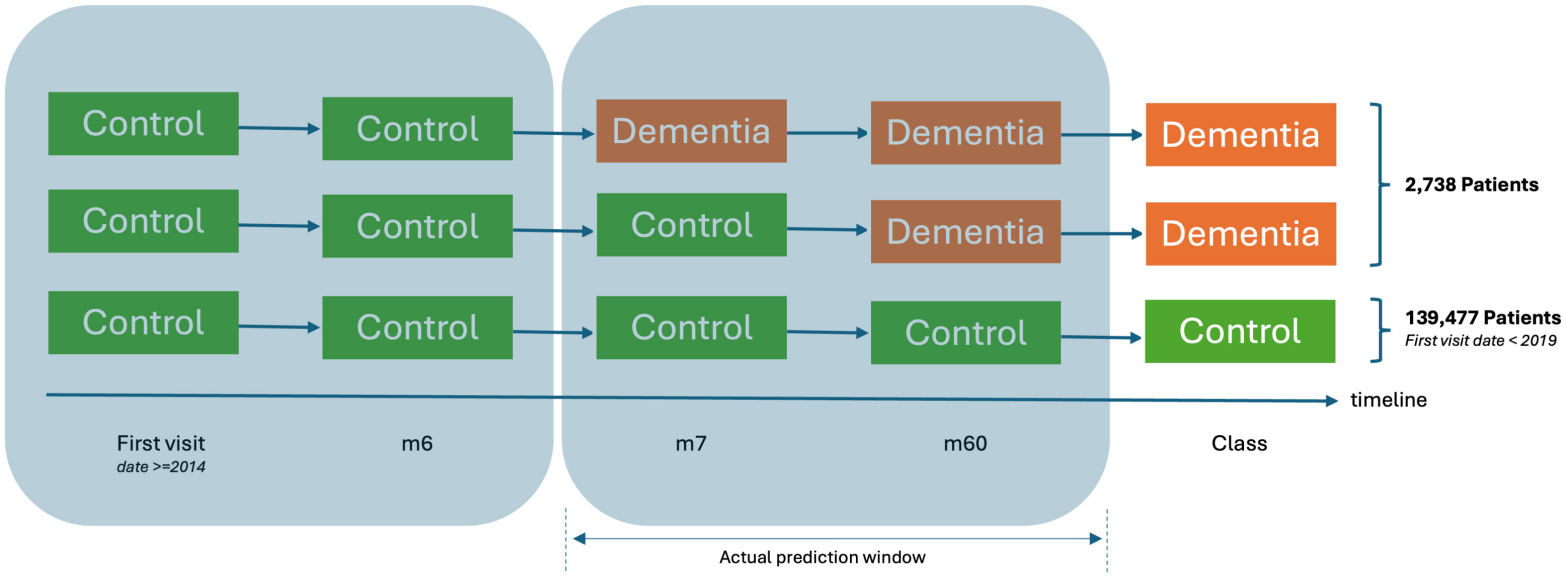
Cohort classification.

The preprocessing phase in prognostic modeling involves detecting, correcting, or eliminating errors, inconsistencies, and inaccuracies within a dataset to enhance its quality and reliability for machine learning models. Additionally, strategies to address imbalances in the dataset are employed to ensure that the model performs effectively across both classes. These methods help mitigate bias and improve the model's ability to generalize to unseen data. Generally, preprocessing guarantees data completeness, accuracy, and consistency, while reducing noise and potential biases in data-driven applications. The dataset showed substantial imbalance, with 2,738 cases comprising just 1.9% of the 139,477 controls (Figure 3). To address this imbalance without reducing the control sample size, we applied the Repeated Random Undersampling Cross-Evaluation method (He and Ma, 2013). Specifically, the control group was divided into 51 equally sized subsets, each matching the size of the minority class. Each subset was then paired with the same minority class to form a balanced training dataset. The model was trained independently on each subset, and the performance metrics were subsequently aggregated.

The model training hyperparameters were tuned separately for each subset, selecting the configuration that achieved the highest mean accuracy across cross-validation folds. Subsequently, each of the two models was standardized using the selected set of hyperparameters. This study utilizes RandomForest and XGBoost algorithms to validate and compare the performance of the applied method.

### 2.5 Missing values

Detecting missing values and determining an appropriate handling strategy, such as removing rows or columns, imputing values, or encoding missingness as a feature, is a key aspect of preprocessing (Ren et al., 2023). Given that the final dataset contained 78% missing values, largely due to the transposition of time-stamped clinical data into a tabular format, features with more than 90% missingness were excluded, reducing the number of effective predictors from 1,193 to 166. This threshold was chosen to reduce model complexity and minimize noise from sparsely observed variables. While some infrequently ordered laboratory tests may be highly informative, an empirical assessment using XGBoost's feature importance measures indicated that features with extremely high missingness contributed minimally to model performance. For the remaining missing values, imputation was not applied, since missing information in clinical data is often not at random (MNAR) and it may carry important clinical meaning leading to biased results when imputing laboratory measurements (Ibrahim et al., 2012; Li et al., 2021).

### 2.6 Model development

We evaluated the performance of two widely used ensemble learning algorithms, Random Forest (RF) and XGBoost (XGB), to tackle the challenges posed by high-dimensional and heterogeneous data commonly found in EHRs, given their well-established robustness and effectiveness in handling complex datasets (Lebedev et al., 2014; Moore and Bell, 2022).

Tree-based ensemble methods such as Random Forest and XGBoost are specifically designed to mitigate overfitting compared with single decision trees. Random Forest achieves this by combining the predictions of many decorrelated decision trees, each trained on bootstrapped samples of the data and with randomized feature selection. This design reduces variance and prevents the model from memorizing noise in the training set, leading to better generalization (Breiman, 2001). Similarly, XGBoost incorporates boosting with regularization techniques (both L1 and L2 penalties) and shrinkage, which prevent the algorithm from fitting noise or spurious patterns in the training data and thereby further reduce the risk of overfitting while maintaining predictive accuracy (Chen and Guestrin, 2016). Both algorithms have been shown in applied studies to achieve high predictive performance while maintaining robustness against overfitting, making them well suited for clinical prediction tasks.

Random Forest and XGBoost showed comparable performance in terms of accuracy and generalization. The objective was to identify a model that most effectively captures the underlying patterns in the data and exhibits strong generalization characteristics. Thus, we carried out a comprehensive evaluation using a 5-repeats, 5-fold Repeated Stratified KFold cross-validation process across all 51 dataset partitions resulting in 1,275 iterations, followed by averaging model prediction performance results on unseen (test) data to assess overall effectiveness and generalization. The training hyperparameters for each model were individually optimized for each of 51 subsets using the Bayesian optimization with cross-validation method (BayesSearchCV), and selecting the configuration that yielded the highest mean accuracy across cross-validation folds. Subsequently, both models were standardized hyperparameter tuning. While both Random Forest and XGBoost are tree-based ensemble methods, Random Forest is often favored for clinical interpretation due to its use of independent decision trees, making it easier to extract and analyze individual trees (Laabs et al., 2024). Additionally, although it averages predictions across multiple trees, each tree remains interpretable on its own.

### 2.7 Decision paths

Supplementary Table 8 presents a set of decision paths (from root to leaf) identified through the ensemble method comprising multiple decision trees of the Random Forest Classifier. The classifier is configured with 100 estimators, meaning each model consists of 100 trees. To evaluate model performance, Repeated Stratified K-Fold Cross-Validation is employed with 5 splits and 5 repeats, resulting in 25 unique train-test splits. For each split, a separate Random Forest model is trained, leading to the generation of 2,500 decision trees across all iterations. The model achieving the highest test F1 score of disease class during cross-validation is selected as the optimal classifier. Subsequently, the 100 decision trees from this best-performing forest are passed individually to a function that transforms each tree into a tabular format and appends them to a dataframe, enabling further selection of the most informative decision pathways based on a set of predefined criteria. These criteria included leaf nodes containing more than 140 samples, with at least 72% classified as disease cases, and an F1 score of disease class exceeding 0.70 for the corresponding tree. To increase the clinical relevance and confidence in our findings, 10 decision pathways were selected (Supplementary Table 8) from a total pool of 5,100 trees for expert interpretation. These rule-based pathways enhance clinical interpretability and offer a transparent, intuitive framework that supports faster and more accurate dementia diagnosis.

For example, the clinical interpretation of the ruleset in Table 4 indicates that among 252 patients with creatinine levels within the normal range but near the upper limit, VLDLCALC values close to the lower bound, weight below 3167.60 oz, a normal CHOL/HDL ratio, low TSH levels, normal (to low) total protein, and low white blood cell counts, 75% developed dementia within 5 years.

**Table 4:** Identifying dementia through diagnostic pathways formed by patterns of risk factors.

**Decision path**
**IF**
CREATININE^a^ < 0.94 AND
VLDLCALC^a^ < 0.10 AND
WEIGHT/SCALE < 3167.60 AND
CHOLHDL^a^ < 0.39 AND
TSH^a^≥0.19 AND
PROT^a^ < 0.47 AND
WBC^a^≥0.21
**THEN** DEMENTIA: 75% Total Samples: 252

^a^Laboratory markers with measurement normalized within the **normal reference range** (NR) Section 2.3.

Unlike black-box models, rule base structure follows a clear, logic that clinicians can easily explain, enhancing trust and usability in practice. They highlight the most informative clinical features and reveal complex interactions between risk factors, which may not be evident through traditional methods. This allows for early detection of dementia by identifying subtle but meaningful patterns. Decision pathways also enable rapid triage and risk stratification, helping clinicians classify patients into risk categories and prioritize timely interventions. Additionally, they can be tailored to align with clinical guidelines, making them highly adaptable for practical use. Overall, the method of rule-based clinical interpretation offers a balance of interpretability, speed, and accuracy that is particularly valuable in managing complex conditions like dementia.

## 3 Results

The performance results for the case and control classes are very similar, as expected in a balanced dataset. The XGBoost model demonstrated performance similar to or slightly better than the Random Forest model (Table 5) with the mean F1-score for the case class in the population ranging from 72.5% to 72.6% for the XGBoost classifier and from 71.4% to 71.5% for the Random Forest. Both models demonstrate AUROC values ranging from 0.77 to 0.79. The 95% Confidence Intervals (CI) for both models were computed using the Student's t-distribution, providing an estimate of the precision around the performance metrics.

**Table 5:** Model prediction and generalization capabilities after 1,275 model iterations.

**Metric**	**Random Forest** ^**a**^		**XGBoost** ^**a**^	
	**Value (Std)**	**95% CI** ^b^	**Value (Std)**	**95% CI** ^b^
Mean AUROC	0.776 (0.014)	0.775–0.777	0.795 (0.014)	0.794–0.795
Mean precision control	0.718 (0.015)	0.718–0.719	0.727 (0.025)	0.726–0.729
Mean precision case	0.698 (0.015)	0.697–0.698	0.718 (0.021)	0.717–0.719
Mean test accuracy	0.707 (0.014)	0.706–0.708	0.722 (0.014)	0.722–0.723
Mean F1-score control	0.699 (0.016)	0.698–0.700	0.719 (0.016)	0.718–0.720
Mean F1-score case	0.715 (0.013)	0.714–0.715	0.725 (0.014)	0.725–0.726

^a^Library: scikit-learn 1.6.1.

^b^95% confidence intervals for mean estimates were calculated based on the t-distribution.

### 3.1 Contribution of predictors to the outcome

**Figure 4:**
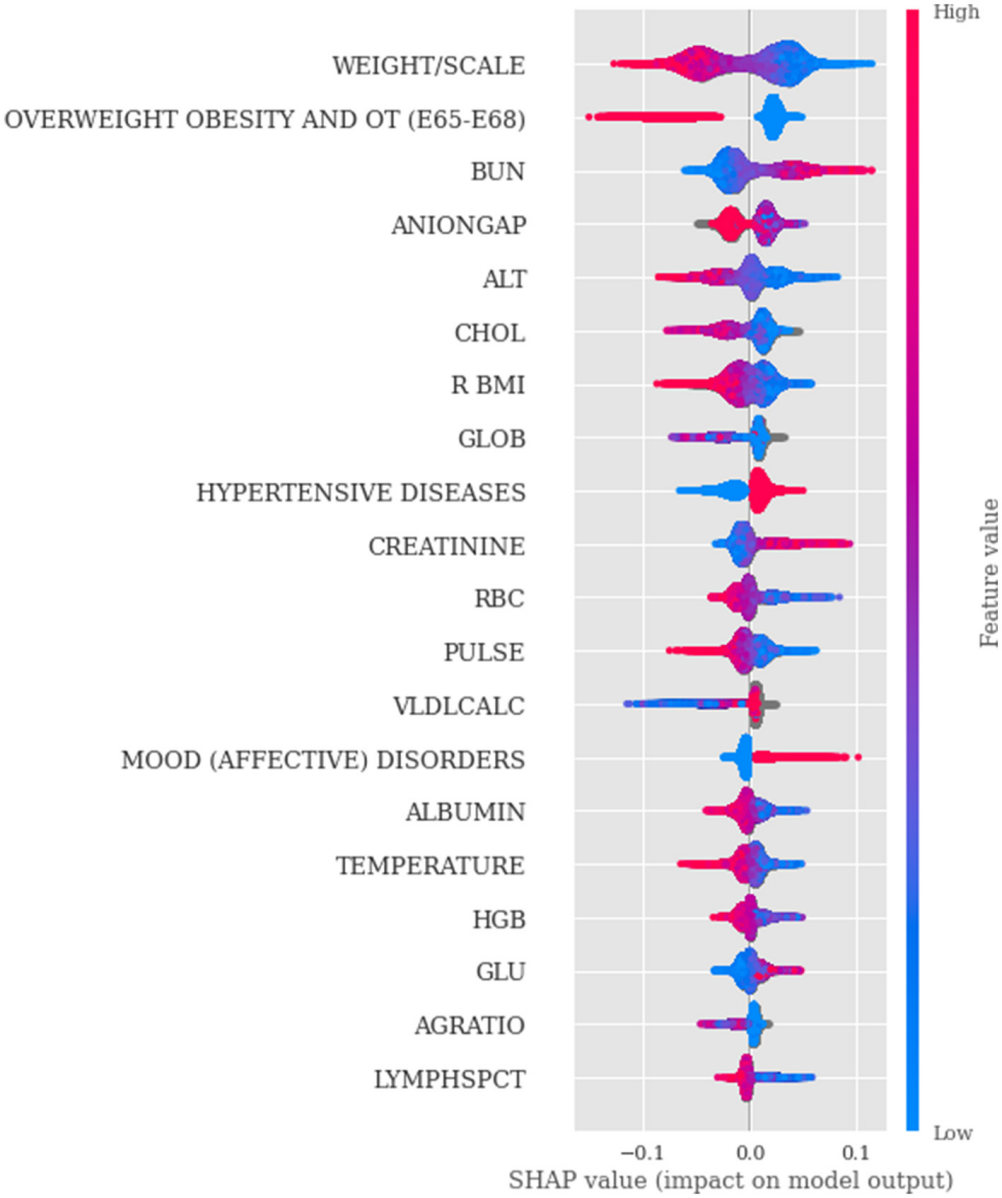
SHAP interpretations obtained from the Random Forest model.

**Figure 5:**
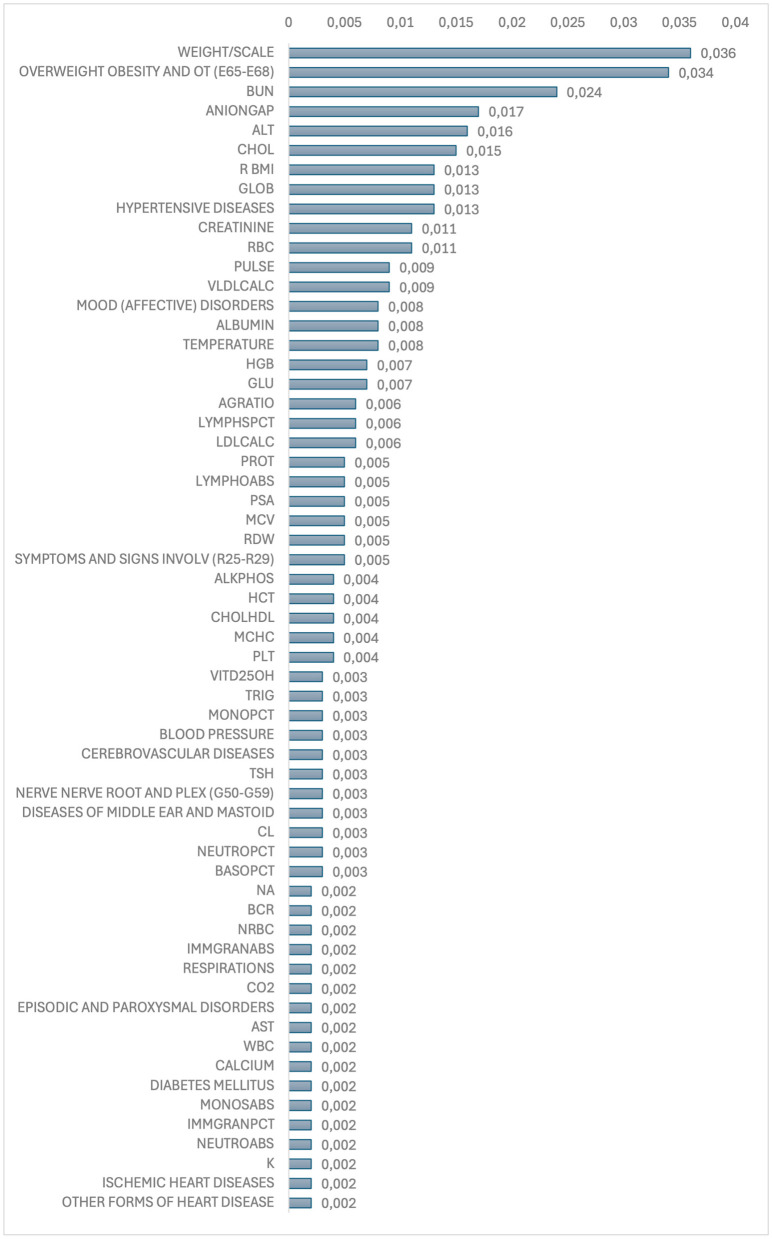
Feature importance.

In machine learning, covariate analysis is crucial for interpreting the influence of features on the target variable, improving model accuracy, and ensuring meaningful insights from the data. The SHAP plot (Figure 4), highlights the top-20 most influential characteristics for the deployed Random Forest model. Given that we adopted a patient-level approach, each dot on the SHAP plot represents an individual patient's clinical measurement (extracted from lab results or vital signs) or diagnosis of comorbidities. The x-axis represents the SHAP value for a specific feature, indicating the impact that feature has on the model's prediction for that patient. The y-axis lists the features (e.g., laboratory results or diagnosis of comorbidities) sorted by their importance in the model while the dots show individual patients' values for that feature and their corresponding SHAP value, which reflects how much that feature contributes to the model's prediction for that patient. The color of the dots indicates the value of the feature for that patient, with the color gradient aiming to illustrate how the feature's value interacts with its impact on the prediction. For the comorbidities (e.g., diagnosis with 1 for True and 0 for False), SHAP values on the x-axis ranged continuously from ± 0.10 since SHAP values represent the impact or contribution of each feature to the model's output (prediction) for each instance, not the binary value itself. Thus, for instance, in the case of HYPERTENSIVE DISEASES, the red color (Dx True) signifies that a prior diagnosis of hypertensive disease is associated with an increased risk of Dementia (positive shap values to the right). The SHAP feature importance (Figure 5) is determined by averaging the absolute SHAP values for the disease class across all samples. This measures the average magnitude of each feature's impact on the model's prediction, regardless of direction. The resulting values indicate the relative contribution of each feature to the prediction for the disease class with higher values signifying greater importance. The remaining features up to 166 exhibit zero SHAP importance, having no measurable impact on the model's predictions.

## 4 Discussion

Our aim was to develop 5-year dementia prediction models using real-world data. The study was based on a single cohort that included outpatients from the memory clinic of the Johns Hopkins Memory and Alzheimer's Treatment Center (JHMATC) in Baltimore, Maryland, USA, as well as outpatients from primary care clinics across the Maryland/DC area.

We evaluated two widely used ensemble methods—Random Forest and XGBoost—to address the challenges of high-dimensional, heterogeneous electronic health record (EHR) data. These algorithms were selected for their ability to model nonlinear relationships and complex interactions among multiple predictors. In future work, we will extend the comparison to include additional approaches such as LASSO and RUSBoost with the same evaluation pipeline.

The SHAP plot (Figure 4), and the feature importance analysis (Figure 5) indicate that a diagnosis of OVERWEIGHT, OBESITY, AND OTHER HYPERALIMENTATION (E65-E68) is associated with a decreased risk of dementia. This finding runs counter to the common hypothesis that midlife obesity increases the possibility of developing dementia in later life. Instead, the evidence suggests that being underweight may be linked to a higher dementia risk. These unexpected findings highlight the necessity for further investigation into the underlying causes and their potential implications for public health (Qizilbash et al., 2015). A similar pattern is observed with other key anthropometric features such as WEIGHT/SCALE and BMI, which are continuous variables derived from the Epic (vital signs) dataset. Other findings of this study suggest that lower values of alanine aminotransferase (ALT), cholesterol (CHOL), and red blood cells (RBCs) are associated with an increased risk of dementia. The Atherosclerosis Risk in Communities (ARIC) Study found that individuals with ALT levels below the 10th percentile had a 34% higher risk of developing dementia compared to those in the second quintile. This association remained significant even after adjusting for factors like age, sex, race, education, and APOE4 genotype. The study suggests that low ALT levels may indicate reduced liver function, which could contribute to cerebral hypometabolism and neurotransmitter impairment, thereby increasing dementia risk (Lu et al., 2021).

Similarly, the study by Wang et al. (2018) demonstrated that over a 7-year follow-up of 1,800 elderly individuals, those with comparatively low serum ALBUMIN levels faced more than double the risk of developing mild cognitive impairment. These findings indicate that albumin may act as an independent risk factor for MCI in the elderly.

Another study showed that involving over 300,000 UK Biobank participants identified that low levels of hemoglobin (HGB) and red blood cell (RBC) distribution width (RDW) were associated with an increased risk of dementia. Specifically, anemia was linked to a 56% higher risk of developing dementia. The study also found that low levels of RBCs and hemoglobin could lead to decreased oxygen-carrying capacity of the blood, contributing to the pathogenesis of dementia (Qiang et al., 2023).

Conversely, there is growing evidence that elevated blood urea nitrogen (BUN) levels (an indicator of kidney dysfunction) are associated with an increased risk of dementia. Mendelian Randomization Study used genetic data to support a causal link between impaired kidney function (including markers like BUN) and increased dementia risk. This strengthens the argument that the relationship is not just correlational (Huang et al., 2024). Similarly, elevated serum CREATININE levels, indicative of reduced kidney function, have been associated with an increased risk of cognitive decline and dementia (Xiao et al., 2023). Diagnosis of HYPERTENSION (high blood pressure) is strongly associated with an increased risk of dementia, including Alzheimer's disease and vascular dementia with numerous large-scale cohort studies to consistently show that individuals with hypertension, especially in midlife, have a significantly higher risk of developing dementia later in life (Kennelly et al., 2009).

When we compared our results with the best-known non-AI approaches in the dementia-risk literature, the performance gap and its source became clearer. Classical mid-life risk scores such as CAIDE, which rely on an additive mix of age, blood pressure, cholesterol, body-mass index and education, consistently discriminate future dementia with AUROC between 0.64 and 0.78 across external validations (Pietilä et al., 2025). Our random forest approach achieved a mean AUROC of 0.776 and a test accuracy of 0.707 which is a modest but consistent improvement that is entirely explained by the model's ability to capture complex high-order interactions. For example, one of the ten decision paths that the model retained identified a 75% risk of developing dementia within 5 years (Table 4). This high-risk pattern appeared in 252 patients who shared seven common clinical findings: creatinine levels near the upper limit, low VLDL cholesterol, below-average weight, a normal cholesterol-to-HDL ratio, low thyroid-stimulating hormone, low total protein, and a reduced white blood cell count. On their own, these values might seem unremarkable or even protective, but together they marked a group at significantly elevated risk. Traditional stepwise regression would have likely missed this pattern, since each variable by itself shows only a weak association with the outcome. This observed performance gain should not be interpreted as a solely endorsement of AI methods over traditional approaches. Rather, it reflects the capacity of ensemble learning to systematically explore high-dimensional interactions and uncover clinically meaningful risk profiles that are likely to remain undetected by conventional statistical models which are limited by predefined terms and a narrow set of interaction terms.

To contextualize our findings within the current landscape of dementia risk modeling, we reviewed several recent, high-quality studies. Schliep et al. (2024) linked 4,206 participants from the Cache County cohort to 163 ICD-coded diagnostic categories and six sociodemographic variables. Using a 1-year prediction horizon, their model achieved an AUROC of 0.67, which increased to 0.77 when dementia was defined directly from ICD codes rather than through adjudication, highlighting the performance limitations imposed by sparse feature sets. Tang et al. (2024) trained a random forest classifier on 749 Alzheimer's cases and 250,545 controls, reporting an AUROC that rose from 0.72 seven years before onset to 0.81 on the index date. Their use of a biomedical knowledge graph further identified hyperlipidemia and osteoporosis as early, sex-specific predictors. The Emergency Department Dementia Algorithm (EDDA), developed from 759,665 ED visits using only a limited set of triage vitals and medication fields, achieved an AUROC of 0.85 on a held-out test set and 0.93 in external validation, demonstrating that highly focused, real-time features can still yield strong short-term predictive performance (Cohen et al., 2025).

Against these benchmarks our approach achieved an AUROC of 0.776 and a test accuracy of 0.707 (Table 5). Notably, SHAP analysis identified renal markers (BUN, creatinine) and lipid-related variables that align with the hyperlipidemia signal described by Tang et al. (2024), while also highlighting low-grade anemia and bilirubin levels which are features that are not detectable in imaging-based approaches such as Eye-AD. These findings suggest that although different modalities capture distinct biological signatures, a comprehensive and widely available EHR feature set can attain performance comparable to or exceeding that of specialized models, while providing additional, clinically interpretable risk factors.

We re-estimated the model using only the most relevant features, specifically those with an importance score greater than or equal to 0.005 (Figure 5), in order to assess how well the model performs under reduced dimensionality. After this reduction, the model was re-evaluated with 27 features instead of the original 166, which led to only an AUROC performance drop of about 3%.

A potential source of bias in our dataset is the use of questionnaire-derived variables, due to self-reported measures that are inherently susceptible to recall bias, response bias, and subjective interpretation, which may introduce variability and affect data reliability. For example, patients may underreport or overreport lifestyle factors, caregivers may provide inconsistent information depending on their perceptions, and responses can vary based on education, cultural background, or cognitive state. These issues can limit accuracy and introduce systematic differences between groups, which in turn may affect predictive modeling if questionnaire-derived variables represent a substantial proportion of the dataset. In our study, however, questionnaire-based data represented only a very small fraction of the features that are used in our models, in contrast to the much larger set of objective measures such as laboratory test results, diagnostic codes, and comorbidities. Because of this imbalance, questionnaire variables had negligible influence on the models' outcomes and did not alter the relative feature contributions. SHAP analysis (Figure 4) confirmed that the main predictors were drawn from objective clinical data. While the potential for bias in self-reported measures is important to acknowledge, in this case their limited role reduces the risk of any meaningful impact on model validity.

The conclusions of this study should be generalized with caution and are most applicable under circumstances where the study population and data characteristics closely resemble those of our cohort. Specifically, the findings are most relevant to populations with similar demographic profiles, clinical features, and healthcare contexts as represented in the dataset. Because dementia is influenced by genetic, social, environmental, and lifestyle factors, the predictive patterns identified here may not hold in populations that differ substantially along these dimensions. Moreover, generalization is most appropriate when the available clinical and demographic variables overlap with those included in our models. Since our predictions are based on a specific set of features, applying these models in settings where such information is incomplete or systematically different may reduce accuracy. Although the SHAP analysis increases transparency by identifying which features most strongly influence predictions, it does not eliminate the risk that other unmeasured factors could play a critical role in dementia risk.

## 5 Conclusions

This study demonstrates the feasibility of using machine learning to predict the risk of Alzheimer's disease and related dementias from real-world EHR data. By transforming time-stamped clinical records into structured predictors, the models achieved strong performance with AUROC values between 0.77 and 0.79. Importantly, our approach goes beyond individual risk factors by identifying combinations of predictors that form clinical pathways, or sets of rules, associated with dementia outcomes. This offers interpretable insights into disease onset and supports more effective risk stratification.

## Data Availability

The data analyzed in this study is subject to the following licenses/restrictions: the data used in this study contain protected patient information and cannot be released publicly. Requests to access these datasets should be directed to kostas@jhmi.edu.

## References

[B1] Akter S. Liu Z. Simoes E. J. Rao P. (2025). Using machine learning and electronic health record (EHR) data for the early prediction of alzheimer's disease and related dementias. J. Prev. Alzheimers Dis. 12:100169. doi: 10.1016/j.tjpad.2025.100169 40246680 PMC12321625

[B2] Al-Sahab B. Leviton A. Loddenkemper T. Paneth N. Zhang B. (2024). Biases in electronic health records data for generating real-world evidence: an overview. J. Healthc. Inf. Res. 8, 121–139. doi: 10.1007/s41666-023-00153-2 38273982 PMC10805748

[B3] Amrollahi F. Shashikumar S. P. Holder A. L. Nemati S. (2022). Leveraging clinical data across healthcare institutions for continual learning of predictive risk models. Sci. Rep. 12:8380. doi: 10.1038/s41598-022-12497-7 35590018 PMC9117839

[B4] Bakouny Z. Patt D. A. (2021). Machine learning and real-world data: more than just buzzwords. JCO Clin. Cancer Inform. 5, 811–813. doi: 10.1200/CCI.21.00092 34383581

[B5] Bastarache L. Brown J. S. Cimino J. J. Dorr D. A. Embi P. J. Payne P. R. . (2022). Developing real-world evidence from real-world data: transforming raw data into analytical datasets. Learn. Health Syst. 6:e10293. doi: 10.1002/lrh2.10293 35036557 PMC8753316

[B6] Boustani M. Perkins A. J. Khandker R. K. Duong S. Dexter P. R. Lipton R. . (2020). Passive digital signature for early identification of alzheimer's disease and related dementia. J. Am. Geriatr. Soc. 68, 511–518. doi: 10.1111/jgs.16218 31784987

[B7] Breiman L. (2001). Random forests. Mach. Learn. 45, 5–32. doi: 10.1023/A:1010933404324

[B8] Chauhan V. K. Thakur A. O'Donoghue O. Rohanian O. Molaei S. Clifton D. A. (2024). Continuous patient state attention model for addressing irregularity in electronic health records. BMC Med. Inform. Decis. Mak. 24:117. doi: 10.1186/s12911-024-02514-2 38702692 PMC11069217

[B9] Chen T. Guestrin C. (2016). “Xgboost: a scalable tree boosting system,” in Proceedings of the 22nd ACM SIGKDD International Conference on Knowledge Discovery and Data Mining, 785–794. doi: 10.1145/2939672.2939785

[B10] Cohen I. Taylor R. A. Xue H. Faustino I. V. Festa N. Brandt C. . (2025). Detection of emergency department patients at risk of dementia through artificial intelligence. Alzheimers Dement. 21:e70334. doi: 10.1002/alz.70334 40457744 PMC12130574

[B11] Collins F. Tabak L. (2014). Using machine learning to identify health outcomes from electronic health record data. Nature 505, 612–613. doi: 10.1038/505612a 24482835 PMC4058759

[B12] DeMers D. Wachs D. (2025). “Physiology, mean arterial pressure,” in StatPearls [Internet] (Treasure Island, FL: StatPearls Publishing). Available online at: https://www.ncbi.nlm.nih.gov/books/NBK538226/30855814

[B13] Ferrao J. C. Oliveira M. D. Janela F. Martins H. M. G. (2017). Preprocessing structured clinical data for predictive modeling and decision support. Appl. Clin. Inform. 7, 1135–1153. 10.4338/ACI-2016-03-SOA-0035 PMC5228148 27924347

[B14] Ford E. Rooney P. Oliver S. Hoile R. Hurley P. Banerjee S. . (2019). Identifying undetected dementia in UK primary care patients: a retrospective case-control study comparing machine-learning and standard epidemiological approaches. BMC Med. Inform. Decis. Mak. 19, 1–9. doi: 10.1186/s12911-019-0991-9 31791325 PMC6889642

[B15] Gao X. R. Chiariglione M. Qin K. Nuytemans K. Scharre D. W. Li Y.-J. . (2023). Explainable machine learning aggregates polygenic risk scores and electronic health records for alzheimer's disease prediction. Sci. Rep. 13:450. doi: 10.1038/s41598-023-27551-1 36624143 PMC9829871

[B16] Hanley J. A. McNeil B. J. (1982). The meaning and use of the area under a receiver operating characteristic (ROC) curve. Radiology 143, 29–36. doi: 10.1148/radiology.143.1.7063747 7063747

[B17] He H. Ma Y. (eds.). (2013). Imbalanced Learning: Foundations, Algorithms, and Applications. John Wiley & Sons: Hoboken, NJ. doi: 10.1002/9781118646106

[B18] Huang H. Ren Y. Wang J. Zhang Z. Zhou J. Chang S. . (2024). Renal function and risk of dementia: a mendelian randomization study. Ren. Fail. 46:2411856. doi: 10.1080/0886022X.2024.2411856 39412044 PMC11485685

[B19] Ibrahim J. G. Chu H. Chen M.-H. (2012). Missing data in clinical studies: issues and methods. J. Clin. Oncol. 30, 3297–3303. doi: 10.1200/JCO.2011.38.7589 22649133 PMC3948388

[B20] Jammeh E. A. Camille B. C. Stephen W. P. Escudero J. Anastasiou A. Zhao P. . (2018). Machine-learning based identification of undiagnosed dementia in primary care: a feasibility study. BJGP Open 2:bjgpopen18X101589. doi: 10.3399/bjgpopen18X101589 30564722 PMC6184101

[B21] Kennelly S. P. Lawlor B. A. Kenny R. A. (2009). Blood pressure and dementia—a comprehensive review. Ther. Adv. Neurol. Disord. 2, 241–260. doi: 10.1177/1756285609103483 21179532 PMC3002634

[B22] Kim M. K. Rouphael C. McMichael J. Welch N. Dasarathy S. (2023). Challenges in and opportunities for electronic health record-based data analysis and interpretation. Gut Liver 18:201. doi: 10.5009/gnl230272 37905424 PMC10938158

[B23] Kim S. Min W.-K. (2025). Toward high-quality real-world laboratory data in the era of healthcare big data. Ann. Lab. Med. 45, 1–11. doi: 10.3343/alm.2024.0258 39344148 PMC11609703

[B24] Laabs B.-H. Westenberger A. König I. R. (2024). Identification of representative trees in random forests based on a new tree-based distance measure. Adv. Data Anal. Classif. 18, 363–380. doi: 10.1007/s11634-023-00537-7

[B25] Lebedev A. Westman E. Van Westen G. Kramberger M. Lundervold A. Aarsland D. . (2014). Random forest ensembles for detection and prediction of alzheimer's disease with a good between-cohort robustness. Neuroimage Clin. 6, 115–125. doi: 10.1016/j.nicl.2014.08.023 25379423 PMC4215532

[B26] Li J. Yan X. S. Chaudhary D. Avula V. Mudiganti S. Husby H. . (2021). Imputation of missing values for electronic health record laboratory data. NPJ Digit. Med. 4:147. doi: 10.1038/s41746-021-00518-0 34635760 PMC8505441

[B27] Li Q. Yang X. Xu J. Guo Y. He X. Hu H. . (2023). Early prediction of alzheimer's disease and related dementias using real-world electronic health records. Alzheimers Dement. 19, 3506–3518. doi: 10.3389/978-2-8325-3897-5 36815661 PMC10976442

[B28] Lu Y. Pike J. R. Selvin E. Mosley T. Palta P. Sharrett A. R. . (2021). Low liver enzymes and risk of dementia: the atherosclerosis risk in communities (ARIC) study. J. Alzheimers Dis. 79, 1775–1784. doi: 10.3233/JAD-201241 33459646 PMC8679120

[B29] Lundberg S. M. Lee S.-I. (2017). “A unified approach to interpreting model predictions,” in Advances in Neural Information Processing Systems, Vol. 30, eds. I. Guyon, U. Von Luxburg, S. Bengio, H. Wallach, R. Fergus, S. Vishwanathan, and R. Garnett (Curran Associates, Inc.), 4765–4774. Available online at: https://proceedings.neurips.cc/paper_files/paper/2017/file/8a20a8621978632d76c43dfd28b67767-Paper.pdf

[B30] Mitchell A. J. Shiri-Feshki M. (2009). Rate of progression of mild cognitive impairment to dementia-meta-analysis of 41 robust inception cohort studies. Acta Psychiatr. Scand. 119, 252–265. doi: 10.1111/j.1600-0447.2008.01326.x 19236314

[B31] Moore A. Bell M. (2022). Xgboost, a novel explainable ai technique, in the prediction of myocardial infarction: a uk biobank cohort study. Clin. Med. Insights Cardiol. 16:11795468221133611. doi: 10.1177/11795468221133611 36386405 PMC9647306

[B32] Nori V. S. Hane C. A. Crown W. H. Au R. Burke W. J. Sanghavi D. M. . (2019). Machine learning models to predict onset of dementia: a label learning approach. Alzheimers Dement. Transl. Res. Clin. Interv. 5, 918–925. doi: 10.1016/j.trci.2019.10.006 31879701 PMC6920083

[B33] Office of the National Coordinator for Health Information Technology (2022). National Trends in Hospital and Physician Adoption of Electronic Health Records. Available online at: https://www.healthit.gov/data/quickstats/national-trends-hospital-and-physician-adoption-electronic-health-records (Accessed September 15, 2025).

[B34] Pietilä E. Löyttyniemi E. Koskinen S. Lehtisalo J. Viitanen M. Rinne J. O. . (2025). Enhancing dementia prediction: a 19-year validation of the caide risk score with insulin resistance and apoe ε4 integration in a population-based cohort. J. Prev. Alzheimers Dis. 12:100034. doi: 10.1016/j.tjpad.2024.100034 39863319 PMC12184024

[B35] Powers D. M. W. (2020). Evaluation: from precision, recall and F-measure to ROC, informedness, markedness and correlation. arXiv preprint arXiv:2010.16061.

[B36] Qiang Y.-X. Deng Y.-T. Zhang Y.-R. Wang H.-F. Zhang W. Dong Q. . (2023). Associations of blood cell indices and anemia with risk of incident dementia: a prospective cohort study of 313,448 participants. Alzheimers Dement. 19, 3965–3976. doi: 10.1002/alz.13088 37102212

[B37] Qizilbash N. Gregson J. Johnson M. E. Pearce N. Douglas I. Wing K. . (2015). Bmi and risk of dementia in two million people over two decades: a retrospective cohort study. Lancet Diabetes Endocrinol. 3, 431–436. doi: 10.1016/S2213-8587(15)00033-9 25866264

[B38] Rashidisabet H. Sethi A. Jindarak P. Edmonds J. Chan R. P. Leiderman Y. I. . (2023). Validating the generalizability of ophthalmic artificial intelligence models on real-world clinical data. Transl. Vis. Sci. Technol. 12, 8–8. doi: 10.1167/tvst.12.11.8 37922149 PMC10629532

[B39] Ren L. Wang T. Seklouli A. S. Zhang H. Bouras A. (2023). A review on missing values for main challenges and methods. Inf. Syst. 119:102268. doi: 10.1016/j.is.2023.102268

[B40] Ren W. Liu Z. Wu Y. Zhang Z. Hong S. Liu H. . (2024). Moving beyond medical statistics: a systematic review on missing data handling in electronic health records. Health Data Sci. 4:176. doi: 10.34133/hds.0176 39635227 PMC11615160

[B41] Schliep K. C. Thornhill J. Tschanz J. T. Facelli J. C. Østbye T. Sorweid M. K. . (2024). Predicting the onset of alzheimer's disease and related dementia using electronic health records: findings from the cache county study on memory in aging (1995–2008). BMC Med. Inform. Decis. Mak. 24:316. doi: 10.1186/s12911-024-02728-4 39468568 PMC11520673

[B42] Shickel B. Tighe P. J. Bihorac A. Rashidi P. (2018). Deep representation learning of patient data from electronic health records (EHR): a systematic review. J. Biomed. Inform. 83, 36–46. 10.1016/j.jbi.2020.103671 PMC11290708 33387683

[B43] Tang A. S. Rankin K. P. Cerono G. Miramontes S. Mills H. Roger J. . (2024). Leveraging electronic health records and knowledge networks for alzheimer's disease prediction and sex-specific biological insights. Nat. Aging 4, 379–395. doi: 10.1038/s43587-024-00573-8 38383858 PMC10950787

[B44] Wang L. Wang F. Liu J. Zhang Q. Lei P. (2018). Inverse relationship between baseline serum albumin levels and risk of mild cognitive impairment in elderly: a seven-year retrospective cohort study. Tohoku J. Exp. Med. 246, 51–57. doi: 10.1620/tjem.246.51 30249938

[B45] World Health Organization (1992). International Statistical Classification of Diseases and Related Health Problems. Tenth Revision (ICD-10). World Health Organization: Geneva.

[B46] Xiao Y. Devakumar V. Xu L. Liu L. Mo H. Hong X. (2023). Elevated serum creatinine levels and risk of cognitive impairment in older adults with diabetes: a nhanes study from 2011–2014. Front. Endocrinol. 14:1149084. doi: 10.3389/fendo.2023.1149084 37900140 PMC10603184

