# Correction: Five-year dementia prediction and decision support system based on real-world data

**DOI:** 10.3389/fnagi.2025.1719723

**Published:** 2025-11-12

**Authors:** Themis P. Exarchos, George A. Dimakopoulos, Konstantinos Lazaros, Marios Krokidis, Aristidis Vrahatis, Gerasimos Grammenos, Antigoni Avramouli, Konstantina Skolariki, Roy Adams, Vasiliki Mahairaki, Esther S. Oh, Jeannie Leoutsakos, Paul B. Rosenberg, Constantine G. Lyketsos, Panagiotis Vlamos

**Affiliations:** 1 Bioinformatics and Human Electrophysiology Laboratory, Department of Informatics, Ionian University, Corfu, Greece; 2 Institute of Digital Biomedicine, Ionian University Research and Innovation Center, Corfu, Greece; 3 Johns Hopkins University School of Medicine, Baltimore, MD, United States; 4 Johns Hopkins Bayview Medical Center, Baltimore, MD, United States

**Keywords:** dementia prediction, Alzheimer's disease, electronic health records, clinical study, cognition, patient-level prediction, real-world data, risk prediction

In the published article, the Supplementary Material documents “Supplementary file 1.pdf” and “Supplementary file 2.pdf” were erroneously published with the original version of this paper. These files have now been removed and replaced with the correct Supplementary Material, “Supplementary Data Sheet 1.pdf”.

The original version of this article has been updated.

