# Supplementary material for: Five-year dementia prediction and decision support system based on real-world data
Source: Front Aging Neurosci. 2025 Sep 30;17:1670609. doi: 10.3389/fnagi.2025.1670609 (PMC12518304; doi:10.3389/fnagi.2025.1670609)
Supplement: Supplementary file 1 [file Data_Sheet_1.pdf]

Appendix

Table A outlines the algorithm used to generate the SHAP summary plot, which visualizes the overall behavior of the model (Figure 4). The algorithm iterates over the 51 data partitions sequentially. For each partition, the model is trained using a Random Forest classifier, and cross-validation is performed to split the data into multiple training and test sets. For each of these splits, the model is trained on the training data, and SHAP values are calculated for the predictions on the test data. These SHAP values are collected along with the corresponding test data. The algorithm processes each split within a partition before moving to the partition. Upon completion of all partitions, it generates a SHAP summary plot to illustrate the model's overall behavior.

To evaluate model performance, we utilize the AUROC and F1-score metrics. The Area Under the Receiver Operating Characteristic Curve (AUROC) is widely used to assess a classifier's ability to generalize in binary classification tasks. It measures how well the model distinguishes between positive and negative classes across all classification thresholds [Hanley and McNeil(1982)]. The F1-score, on the other hand, combines precision and recall into a single metric, reflecting the model's effectiveness in correctly identifying the positive class while reducing both false positives and false negatives [Powers and Ailab(2011)].

To explore feature importance we use SHAP (SHapley Additive exPlanations) values. The SHAP method estimates the impact of predictors on each individual prediction, providing a more comprehensive view of feature importance within the model [Lundberg and Lee(2017)]. The SHAP value indicates how much this feature shifts the model's prediction compared to the baseline (the average model output). A SHAP value can be positive or negative depending on whether the feature (True/1 or False/0) pushes the prediction higher or lower. We generated the SHAP plot for the RF model deployment (Figure 4) using Repeated Stratified KFold cross-validation and aggregating SHAP values across test folds for all the 51 partitions to create a comprehensive SHAP plot (Table A).

Table B presents a glossary of comorbidity features, including detailed descriptions of each diagnosis along with the corresponding ICD-10 code ranges that were used in this study. The list of comorbidity ICD-10 codes is derived from the International Classification of Diseases catalog [World Health Organization(1992)]. In International Statistical Classification of Diseases and Related Health Problems, Tenth Revision published by the World Health Organization (WHO), the classification is organized hierarchically into multiple levels. The classification consist of 22 chapters, each representing a major disease category (e.g., Chapter I: Certain infectious and parasitic diseases). Each chapter is divided into blocks (ICD-10 range) of related (or similar) conditions (e.g., N17-N19: ACUTE KIDNEY FAILURE AND CHRONIC KIDNEY DISEASE). In the dataset, diagnoses were assigned at the block level, with patients labeled as "True" if a diagnosis was present, and "False" otherwise. For example, if a patient had a recorded encounter with a diagnosis of E66.0 (obesity due to excess calories), the block level "OVERWEIGHT, OBESITY AND OTHER HYPERALIMENTATION (E65-E68)" is assigned a "True" diagnosis.

Table A: SHAP Summary Plot Computation

```
idx ← 0        # Initialize partition index
shap_values_all ← empty        # Array initialization
X_test_df ← empty        # Dataframe initialization
while idx < 52 do        # Iterate over 51 partitions
    X ←Partition[idx]
    y ←Target[idx]
    clf ← RandomForestClassifier(params)
    rskf ← RepeatedStratifiedKFold(n_splits = 5, n_repeats = 5)
    for all train_index, test_index in rskf.split(X, y) do
        X_train, X_test ← X[train_index], X[test_index]
        y_train, y_test ← y[train_index], y[test_index]
        clf.fit(X_train, y_train)
        explainer ← shap.TreeExplainer(clf)
        shap_vals ← explainer.shap_values(X_test)
        Append X_test to X_test_df
        Append shap_vals to shap_values_all
    end for
    idx ← idx + 1
end while
Display shap.summary_plot(shap_values_all[1], X_test_df)
```

Process to compute the SHAP summary plot for model interpretation

10.3389/fnagi.2025.1670609

Table B: Glossary of comorbidities

Diagnosis	ICD-10 range[1]
ACUTE KIDNEY FAILURE AND CHRONIC KIDNEY DISEASE	N17-N19
BENIGN NEOPLASMS, EXCEPT BENIGN NEUROENDOCRINE TUMORS	D10-D36
DISORDERS OF BONE DENSITY AND STRUCTURE	M80-M85
MENTAL AND BEHAVIORAL DISORDERS DUE TO PSYCHOACTIVE SUBSTANCE USE	F10-F19
NERVE, NERVE ROOT AND PLEXUS DISORDERS	G50-G59
NONINFLAMMATORY DISORDERS OF FEMALE GENITAL TRACT	N80-N98
OTHER DISEASES OF THE URINARY SYSTEM	N30-N39
OTHER DISEASES OF UPPER RESPIRATORY TRACT	J30-J39
OTHER DISORDERS OF THE SKIN AND SUBCUTANEOUS TISSUE	L80-L99
OVERWEIGHT, OBESITY AND OTHER HYPERALIMENTATION	E65-E68
SLIPPING, TRIPPING, STUMBLING AND FALLS	W00-W19
SYMPTOMS AND SIGNS INVOLVING THE CIRCULATORY AND RESPIRATORY SYSTEMS	R00-R09
SYMPTOMS AND SIGNS INVOLVING THE DIGESTIVE SYSTEM AND ABDOMEN	R10-R19
SYMPTOMS AND SIGNS INVOLVING THE NERVOUS AND MUSCULOSKELETAL SYSTEMS	R25-R29
SYMPTOMS AND SIGNS INVOLVING THE SKIN AND SUBCUTANEOUS TISSUE	R20-R23
VIRAL INFECTIONS CHARACTERIZED BY SKIN AND MUCOUS MEMBRANE LESIONS	B00-B09

[1] International Classification of Diseases [World Health Organization(1992)]

Table 8: Optimal pathways using patterns of risk factors for dementia clinical diagnosis

IF:<br>CREATININE[1] $<$ 0.94 AND<br>VLDLCALC[1] $<$ 0.10 AND<br>WEIGHT/SCALE $<$ 3167.60 AND<br>CHOLHDL[1] $<$ 0.39 AND<br>TSH[1] $\geqslant$ 0.19 AND<br>PROT[1] $<$ 0.47 AND<br>WBC[1] $\geqslant$ 0.21<br>THEN DEMENTIA: 75% Total Samples: 252
IF:<br>GLOB[1] $<$ 0.05 AND<br>WEIGHT/SCALE $<$ 3163.40 AND<br>BUN[1] $\geqslant$ 0.55 AND<br>MCHC[1] $<$ 0.50 AND<br>MPV[1] $<$ -0.02 AND<br>PLT[1] $<$ 0.16<br>THEN DEMENTIA: 73% Total Samples: 213

10.3389/fnagi.2025.1670609

10.3389/fnagi.2025.1670609

Optimal pathways using patterns of risk factors for dementia clinical diagnosis (continued)

IF:
(not) OVERWEIGHT, OBESITY AND OTHER HYPERALIMENTATION (E65-E68) AND
HGB[1] < 0.28 AND
HYPERTENSIVE DISEASES AND
PSA[1] ⩾ 1.40 AND
TRIG[1] ⩾ 0.69 AND
BUN[1] ⩾ 0.38 AND
MCV[1] ⩾ 0.16 AND
R BMI ⩾ 23.05 AND
K[1] < 0.63 AND
NEUTROPCT[1] < 0.99
THEN DEMENTIA: 84% Total Samples: 182

10.3389/fnagi.2025.1670609

Optimal pathways using patterns of risk factors for dementia clinical diagnosis (continued)

IF:

CHOL[1]<0.88 AND
PLT[1]≥0.24 AND
ALT[1]≥0.31 AND
(not) DISEASES OF MIDDLE EAR AND MASTOID AND
CREATININE[1]<1.17 AND
GLOB[1]<-0.15 AND
NEUTROABS[1]≥0.24 AND
RBC[1]≥0.03 AND
(not) VIRAL INFECTIONS CHARACTERIZED BY SKIN AND MUCOUS MEMBRANE LESIONS (B00-B09) AND
BCR[1]≥1.13 AND
(not) OVERWEIGHT, OBESITY AND OTHER HYPERALIMENTATION (E65-E68) AND
METABOLIC DISORDERS AND
ALKPHOS[1]<0.78 AND
HYPERTENSIVE DISEASES AND
(not) MENTAL AND BEHAVIORAL DISORDERS DUE TO PSYCHOACTIVE SUBSTANCE USE (F10-F19) AND
RESPIRATIONS≥16.25

THEN DEMENTIA: 73% Total Samples: 166

10.3389/fnagi.2025.1670609

Optimal pathways using patterns of risk factors for dementia clinical diagnosis (continued)

IF:
ALT[1]<0.52 AND
RBC[1]<0.21 AND
CREATININE[1]⩾0.70 AND
GLOB[1]<0.11 AND
AST[1]<0.66 AND
CALCIUM[1]<0.72 AND
BLOOD PRESSURE<96.50 AND
CL[1]⩾0.28
THEN DEMENTIA: 84% Total Samples: 165

IF:
(not) OVERWEIGHT, OBESITY AND OTHER HYPERALIMENTATION (E65-E68) AND
CREATININE[1]⩾0.56 AND
LDLCALC[1]<0.95 AND
WEIGHT/SCALE<2436.20 AND
PROT[1]⩾0.41 AND
PULSE OXIMETRY<96.25
THEN DEMENTIA: 72% Total Samples: 158

IF:
CREATININE[1]<0.84 AND
WEIGHT/SCALE<2907.60 AND
GLOB[1]<0.04 AND
TEMPERATURE<97.78 AND
PROT[1]<0.36
THEN DEMENTIA: 75% Total Samples: 156

Optimal pathways using patterns of risk factors for dementia clinical diagnosis (continued)

IF:
- GLOB[1] ⩾0.88 AND
- (not) OTHER VIRAL DISEASES AND
- NEUTROABS[1] <0.30 AND
- PSA[1] ⩾1.37 AND
- WEIGHT/SCALE<3027.00 AND
- ALBUMIN[1] ⩾0.42 AND
- (not) NERVE, NERVE ROOT AND PLEXUS DISORDERS (G50-G59) AND
- VITD25OH[1] ⩾0.07 AND
- ALKPHOS[1] ⩾0.25 AND
- CL[1] ⩾-0.22 AND
- MPV[1] ⩾0.96 AND
- ALT[1] <0.40

THEN DEMENTIA: 82% Total Samples: 152

IF:
- ALT[1] <0.50 AND
- RBC[1] <0.23 AND
- R BMI<41.35 AND
- ALT[1] ⩾0.04 AND
- ANIONGAP[1] <1.09 AND
- HYPERTENSIVE DISEASES AND
- AST[1] ⩾0.48 AND
- NEUTROPCT[1] ⩾0.42 AND
- HGBA1C[1] ⩾0.82

THEN DEMENTIA: 90% Total Samples: 149

10.3389/fnagi.2025.1670609

10.3389/fnagi.2025.1670609

Optimal pathways using patterns of risk factors for dementia clinical diagnosis (continued)

IF:
WEIGHT/SCALE<2908.00 AND
GLOB[1]<0.06 AND
RESPIRATIONS⩾16.25 AND
PULSE<92.75 AND
TSH[1]⩾0.20 AND
WBC[1]<0.63
THEN DEMENTIA: 86% Total Samples: 140

Notes:

- WEIGHT/SCALE is measured in **ounces (oz)**
- Blood pressure values were converted into a single numerical metric: the **Mean Arterial Pressure** [DeMers and Wachs(2025)]
- Laboratory markers with superscript[1] indicate that measurement is normalized within **Normal Reference Range**

Examples:

Value ⩾ 1 → measurement is normal or above normal

Value ⩽ 0 → measurement is normal or below normal

Value > 0.6 → measurement is normal close to mean value (0.5)

Value > 0.9 → measurement is (nearly) normal close to upper limit

Value > 0.1 → measurement is (nearly) normal close to lower limit

References

[DeMers and Wachs(2025)] [Dataset] DeMers, D. and Wachs, D. (2025). Physiology, mean arterial pressure. https://www.ncbi.nlm.nih.gov/books/NBK538226/. StatPearls [Internet]. Treasure Island, FL: StatPearls Publishing

[Hanley and McNeil(1982)] Hanley, J. A. and McNeil, B. J. (1982). The meaning and use of the area under a receiver operating characteristic (roc) curve. *Radiology* 143, 29–36

[Lundberg and Lee(2017)] Lundberg, S. M. and Lee, S.-I. (2017). A unified approach to interpreting model predictions. *Advances in neural information processing systems* 30

[Powers and Ailab(2011)] Powers, D. and Ailab (2011). Evaluation: From precision, recall and f-measure to roc, informedness, markedness & correlation. *J. Mach. Learn. Technol* 2, 2229–3981. doi:10.9735/2229-3981

[World Health Organization(1992)] World Health Organization (1992). *International Statistical Classification of Diseases and Related Health Problems. Tenth Revision (ICD-10)* (Geneva: World Health Organization)
